# The NO Pathway as a Target in Patients with Stable and Advanced Heart Failure: An Additional Arrow in Our Quiver!

**DOI:** 10.3390/biom15101420

**Published:** 2025-10-06

**Authors:** Saverio D’Elia, Carmine Gentile, Achille Solimene, Rosa Franzese, Ettore Luisi, Antonio Caiazzo, Luigi Marotta, Simona Covino, Francesco Natale, Francesco S. Loffredo, Paolo Golino, Giovanni Cimmino

**Affiliations:** 1 Cardiology Unit, Azienda Ospedaliera Universitaria Luigi Vanvitelli, 80138 Naples, Italy; saverio.delia@unicampania.it; 2 Department of Advanced Medical and Surgical Sciences, University of Campania “Luigi Vanvitelli”, Piazza Luigi Miraglia, 2, 80138 Naples, Italy; 3 Department of Translational Medical Sciences, Section of Cardiology, University of Campania Luigi Vanvitelli, 80138 Naples, Italy; carmine.gentile16@gmail.com (C.G.); achille.solimene@unicampania.it (A.S.); rosa-franzese@libero.it (R.F.); ettore.luisi@unicampania.it (E.L.); acaiazzo1000@gmail.com (A.C.); marotta.luigi.lm@gmail.com (L.M.); simona.covino97@gmail.com (S.C.); francesco.loffredo@unicampania.it (F.S.L.); paolo.golino@unicampania.it (P.G.); 4 Department of Life Science, Health, and Health Professions, Link Campus University, 00165 Rome, Italy; natalefrancesco@hotmail.com; 5 Vanvitelli Cardiology and Intensive Care Unit, Monaldi Hospital, 80131 Naples, Italy

**Keywords:** nitric oxide, soluble guanylate cyclase, heart failure, endothelial dysfunction, inflammation, vasodilation, myocardial fibrosis, pulmonary hypertension, advanced heart failure, therapeutic targets

## Abstract

The nitric oxide (NO) pathway is a fundamental regulator of vascular tone, myocardial function, and inflammation. In heart failure (HF), especially in advanced stages, dysregulation of NO–soluble guanylate cyclase (sGC)–cyclic guanosine monophosphate (cGMP) signaling contributes to endothelial dysfunction, increased vascular resistance, myocardial fibrosis, and impaired cardiac performance. Chronic inflammation further reduces NO bioavailability, exacerbating HF progression This review synthesizes current knowledge on the role of the NO pathway in HF pathophysiology, with a focus on stable and advanced HF. Special attention is given to patient subgroups with comorbidities such as chronic kidney disease, where modulation of NO signaling may be particularly beneficial. We also evaluate therapeutic strategies targeting NO bioavailability and sGC stimulation. Evidence shows that impaired NO signaling promotes systemic and pulmonary vasoconstriction, elevates ventricular afterload, and worsens cardiac remodeling. Pharmacological agents that restore NO levels or activate downstream effectors such as sGC improve vasodilation, reduce fibrosis, and enhance myocardial relaxation. These effects are especially relevant in advanced HF patients and those with renal impairment, who often exhibit limited responses to conventional therapies. The NO pathway represents a promising therapeutic target in both stable and advanced HF. Modulating this pathway could improve outcomes, particularly in complex populations with multiple comorbidities, highlighting the need for further clinical research and tailored treatments.

## 1. Introduction

### 1.1. Hystorical Background

Nitric oxide (nitrogen monoxide, NO) was first discovered by the English chemist Joseph Priestley in 1772, but its biological significance remained unknown for over two centuries.

In 1977, pharmacologist and physician Ferid Murad demonstrated that nitric oxide is a crucial biological mediator. His studies revealed that organic nitrates, such as nitroglycerin, exert their vasodilatory effect by releasing NO. This discovery represented a pivotal step in understanding the role of NO as a signaling molecule in the cardiovascular system [1].

In 1980, while studying vascular function, Robert F. Furchgott identified a mysterious signaling molecule that relaxed blood vessels and he initially named it “Endothelium-Derived Relaxing Factor” (EDRF) [2], in line with the research conducted by L. J. Ignarro and his team [3]. It was discovered that EDRF was, in fact, nitric oxide. In 1998, Robert F. Furchgott, Louis J. Ignarro, and Ferid Murad were awarded the Nobel Prize in Physiology or Medicine for their pioneering work.

### 1.2. Nitric Oxide Synthesis and Isoforms

NO is a free radical and lipophilic gas capable of easily crossing the cell membrane. It is derived from the amino acid L-arginine and, due to its short half-life, acts within seconds (t^½^ 3–5 s), rapidly degrading into nitrites, nitrates, hydroxyl, and other catabolic derivatives. This very short half-life confines nitric oxide activity to its site of production, preventing long-range diffusion and ensuring highly localized and precise signaling. Such spatial restriction is essential for maintaining its physiological functions, particularly in vascular tone regulation and platelet aggregation [4]. NO regulates a vast array of biological processes within the human body. It is synthesized from L-arginine by a family of enzymes known as nitric oxide synthases (NOSs) [3,4]. The availability of L-arginine can limit NO production, and endogenous inhibitors such as asymmetric dimethylarginine (ADMA) compete with L-arginine for NOS binding, reducing NO synthesis. There are three isoforms of nitric oxide synthase: endothelial NOS (eNOS or NOS3), neuronal NOS (nNOS or NOS1), and inducible NOS (iNOS or NOS2). Their nomenclature is based on the tissue in which they were first identified.

The first two isoforms are constitutive and calcium-dependent, as their activation requires the calcium/calmodulin complex. Intracellular calcium binds to calmodulin (CaM), inducing a conformational change that activates it. Subsequently, activated calmodulin binds to NOS, enabling its enzymatic activity. There are various mechanisms which lead to an increase in intracellular calcium levels. Specifically, in neurons, this occurs through excitatory neurotransmitters, such as glutamate, which activate N-metil-D-aspartato (NMDA) receptors [5]. These receptors are ligand-gated ion channels that, once opened, allow calcium ions to flow into the cell, thereby coupling synaptic activity to nitric oxide production.

These receptors function as ion channels, allowing calcium influx into the cell. In endothelial cells, calcium elevation is mediated by several stimuli, including acetylcholine, bradykinin, or shear stress induced by blood flow.

### 1.3. Mechanisms of Action, Physiological Functions

The inducible isoform (iNOS), predominantly expressed in macrophages, is less sensitive to Ca^2+^ and can increase its synthesis more than 1000-fold in response to inflammatory stimuli such as bacterial lipopolysaccharide (LPS) and cytokines (interleukin-1, IFN-γ) [6]. The gaseous signaling molecule nitric oxide binds to soluble guanylate cyclase (sGC), a heterodimeric enzyme containing a heme protoporphyrin-IX domain, and activates it. This enzyme catalyzes the conversion of guanosine 5′-triphosphate (GTP) into guanosine 3′:5′-cyclic monophosphate (cGMP), thereby increasing its intracellular levels [1,7] cGMP is also synthesized by particulate guanylate cyclase (pGC), a membrane-bound isoform that is typically activated upon binding to specific ligands, such as natriuretic peptides [8].

As an intracellular second messenger, cGMP is degraded by phosphodiesterases (PDEs) [9]. In the vascular system, the effects of cGMP are primarily mediated by cGMP-dependent protein kinase (protein kinase G, PKG). In the vascular smooth muscle cells, PKG activation by cGMP induces vasodilation through multiple mechanisms. PKG can decrease intracellular calcium concentrations, thereby reducing muscle contraction by inhibiting calcium release from intracellular stores and blocking calcium influx through voltage-gated Ca^2+^ channels.

Phosphorylation of phospholamban due to PKG, a key regulator of the sarcoplasmic reticulum Ca^2+^ pump, accelerates Ca^2+^ reuptake into intracellular stores [10]. PKG also contributes to calcium level reduction by opening potassium channels, leading to cellular membrane hyperpolarization [11]. This process results in the closure of L-type calcium channels, further limiting calcium influx into the cell.

In addition, PKG enhances vasodilation by modulating myosin light chain phosphatase (MLCP) activity. This regulation is not direct but occurs through phosphorylation of an inhibitory protein (CPI-17) [11]. When phosphorylated by PKG, CPI-17 is inactivated, relieving its suppression of MLCP. As a result, MLCP becomes active and dephosphorylates myosin light chains, preventing actin–myosin interaction and promoting smooth muscle relaxation, inducing vasodilation.

Moreover, PKG inhibits myosin light chain kinase (MLCK), further decreasing myosin phosphorylation and its binding capacity to action [12,13].

### 1.4. Role in Nervous and Immune Systems

**Figure 1:**
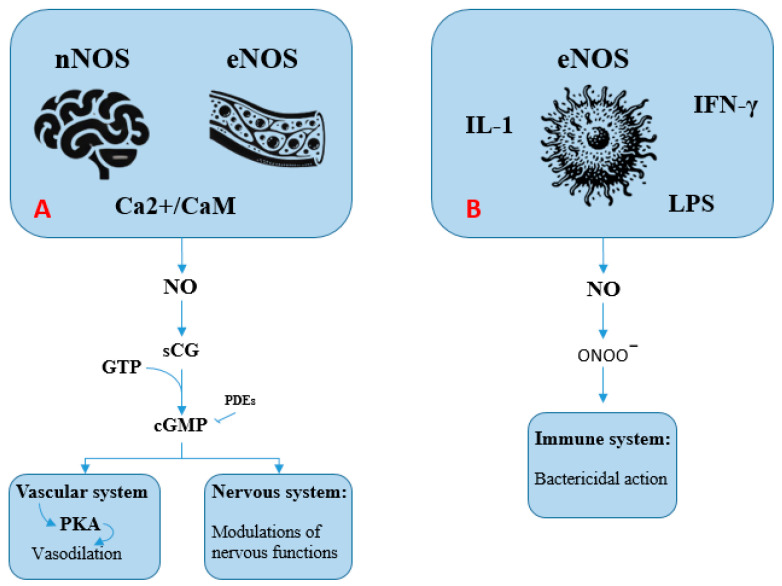
The nitric oxide (NO) pathway (see text for explanation). (**A**): Activation of neuronal (nNOS) and endothelial (eNOS) nitric oxide synthases occurs via the Ca^2+^/calmodulin complex. These enzymes generate NO, which subsequently activates soluble guanylate cyclase (sGC). This enzyme catalyzes the conversion of guanosine 5′-triphosphate (GTP) into guanosine 3′,5′-cyclic monophosphate (cGMP). Acting as a second messenger, cGMP mediates vasodilation in blood vessels and modulates various neuronal functions. (**B**): Inducible nitric oxide synthase (iNOS) is activated in response to inflammatory stimuli, including bacterial lipopolysaccharides (LPS) and cytokines such as interleukin-1 and interferon-gamma (IFN-γ). The NO produced in this context reacts to form compounds such as peroxynitrite (ONOO^−^), which contributes to pathogen destruction through oxidative damage.

NO is a signaling molecule involved in numerous physiological processes beyond vasodilation. In neuronal cells, NO primarily functions as an atypical neurotransmitter, modulating synaptic plasticity, neuronal transmission, and memory. In this context, NO activates soluble guanylate cyclase (sGC), leading to an increase in intracellular cGMP levels, which serves as a second messenger. cGMP, in turn, regulates ion channels, protein kinases, and PDEs, thereby influencing neurotransmission and cerebral blood flow regulation [14]. However, under conditions of oxidative stress or neurodegeneration, excessive NO production can lead to the formation of reactive nitrogen species (RNSs), contributing to neuronal damage. In the immune system, NO plays a crucial role in inflammatory responses and pathogen defense. Activated macrophages produce NO through the inducible nitric oxide synthase (iNOS) enzyme to eliminate bacteria, viruses, and tumor cells. In this case, NO’s effects are not primarily mediated by cGMP but rather by its ability to interact with proteins and lipids, forming reactive compounds such as peroxynitrite (ONOO^−^), which damages pathogens [15]. However, uncontrolled NO production can contribute to pathological processes, including chronic inflammation and tissue damage in autoimmune diseases. A schematic view of the NO pathway is provided in Figure 1.

NO is undoubtedly one of the most versatile and essential signaling molecules in human physiology. From regulating vascular tone and neurotransmission to modulating immune responses, NO plays a fundamental role in maintaining homeostasis across multiple organ systems. As Dr. Jonathan Stamler of Duke University aptly stated: “It does everything, everywhere. There isn’t a major cellular response or physiological effect where nitric oxide isn’t involved. It regulates brain function, airway relaxation, heart rhythm, blood vessel dilation, immune responses, and even limb movement”. This widespread influence highlights NO as a key player in both health and disease, making it a critical target for future research and therapeutic advancements.

## 2. Nitric Oxide and Heart Failure: A Physiological Basis for Clinical Benefits

HF is a heterogeneous clinical syndrome characterized by significant morbidity and mortality, as well as impaired functional capacity and quality of life. The global prevalence of HF is estimated to range between 1% and 3% of the total population [16]. As a consequence, efforts to mitigate its social and economic burden have become a major global public health priority. This has led to a sustained focus within scientific research on the development of novel therapeutic strategies aimed at reducing hospitalization rates and mortality among patients affected by this complex clinical condition.

In HF, chronic inflammation and endothelial dysfunction have been demonstrated to cause alterations in the NO-sGC-cGMP pathway. As such, this pathway represents a critical pathophysiological target that might offer protection against ongoing cardiac damage [17]. The hemodynamic effects resulting from nitric oxide pathway dysregulation in heart failure are multifaceted. Reduced NO bioavailability induces systemic [18] and pulmonary [19] vasoconstriction, which consequently increases left and right ventricular afterload, respectively. Furthermore, impaired endothelium-dependent vasodilatory mechanisms at the coronary level may compromise myocardial perfusion [20]. Nitric oxide also plays a pivotal role in regulating myocardial contractility. Endothelium-derived nitric oxide diffuses into smooth muscle cells, stimulating the production of cGMP, which promotes smooth muscle relaxation and exhibits antiproliferative effects. These mechanisms collectively influence myocardial performance [21]. NO has been shown to exert antifibrotic and antihypertrophic effects by antagonizing endothelin-1, angiotensin II, and aldosterone. Consequently, reduced NO bioavailability permits these molecules to exert their profibrotic effects, promoting interstitial fibrosis [22].

A study demonstrated that reduced NO bioavailability plays a crucial role in the development of heart failure with preserved ejection fraction (HFpEF). Interstitial fibrosis, alongside macro- and microscopic hypertrophy [23,24], contributes to diastolic dysfunction, which is classically manifested as delayed myocardial relaxation and decreased compliance due to increased cardiomyocyte stiffness. On the other hand, other studies [25] have highlighted that intracoronary infusion of sodium nitroprusside results in a reduction in left ventricular (LV) peak systolic pressure, acceleration of LV relaxation, and an improvement in myocardial distensibility.

The NO-sGC-cGMP pathway is also involved in the regulation of sarcomeric protein titin phosphorylation. Specifically, increased cGMP production induces titin phosphorylation through protein kinase G, which enhances the cardiac index and attenuates left ventricular remodeling [26].

Finally, dysregulation of the NO-sGC-cGMP pathway contributes to the development of pulmonary hypertension [27]. Dysregulated smooth muscle tone and pulmonary vascular remodeling lead to increased right ventricular afterload, which, over time, may result in eccentric hypertrophy, uncoupling, and both systolic and diastolic dysfunction [28]. sGC stimulators may act to increase cGMP levels and potentially ameliorate these mechanisms [29].

In advanced HF (AHF) phenotypes—characterized by persistent congestion, low output, and end-organ dysfunction—severe endothelial dysfunction and oxidative stress further reduce NO availability and disrupt cGMP signaling. In these patients, therapeutic strategies that enhance the NO pathway, such as inorganic nitrates/nitrites or sGC stimulators like vericiguat, may offer hemodynamic benefits by reducing afterload, improving renal perfusion, and attenuating myocardial remodeling [30,31]. Recent experimental models have also shown that nitrite–hydralazine combinations exert synergistic antifibrotic and vasodilatory effects, representing a low-cost adjunctive strategy that is potentially useful in advanced or therapy-resistant HF [32,33].

Nitric oxide (NO) also acts as a potent inhibitor of platelet aggregation through the activation of soluble guanylate cyclase, leading to an increase in intracellular cyclic GMP (cGMP) levels and subsequent activation of protein kinase G (PKG). This cascade reduces cytosolic calcium and prevents platelet activation [34]. Recent clinical data further support this effect: perindopril therapy in essential hypertensive patients was shown to reduce adrenaline-induced platelet aggregation over one month of treatment, suggesting ACE inhibitors can enhance NO-mediated anti-aggregatory function in vivo.

Hydrogen sulfide (H_2_S) donors, such as N-acetylcysteine (NAC), enhance the anti-aggregatory effects of NO. A recent study [35] demonstrated that, during coronary artery spasm (CAS), platelet NO signaling is impaired and that NAC, through the release of H_2_S, improves platelet responsiveness to NO. Moreover, combined treatment with nitroglycerin and NAC strengthens the hemodynamic and anti-aggregatory effects of nitroglycerin, lowering the risk of acute myocardial infarction in unstable angina [36]. Angiotensin-converting enzyme (ACE) inhibitors, particularly those containing thiol groups such as captopril, may further potentiate the anti-aggregatory effect of NO and H_2_S donors, likely through the reduction in oxidative stress and the consequent increase in NO bioavailability. The effect of ACE inhibition (e.g., perindopril) in subjects with essential hypertension has been confirmed: after one month of therapy, there was a noticeable decrease in platelet aggregation induced by adrenaline, supporting the conclusion that ACE inhibitors enhance NO’s anti-aggregatory capacity in clinical populations [37].

### Inflammation and the NO Pathway in Heart Failure

Endothelial inflammation has a direct impact on the NO pathway. The release of inflammatory cytokines, such as IL-1β, TNF alpha or INF-γ, physiologically induces the expression of inducible nitric oxide synthases (iNOS), which aim to increase NO production and improve the inflammatory response and defense against possible pathogens [38]. Conversely, the pro-inflammatory state also elicits the production of reactive oxide and nitrogen species that reduce NO bioavailability in endothelial cells and reduce sGC production. On the one hand, the whole process is self-enhanced by the upregulation of cyclo-oxygenase (COX) enzymes, which maintain the inflammatory state [39]; on the other hand, the inflammatory state induces uncoupling of eNOS, with a further reduction in NO bioavailability [40]. The role of inflammation in the pathogenesis of chronic heart failure has been widely investigated and includes both NO-related and non-NO-related processes. 

In heart failure with reduced ejection fraction (HFrEF), secondary to ischemic or non-ischemic cardiac injury, not only does IL-1 signaling determine iNOS-related damage but it also directly induces inhibition of L-type calcium channel, beta-adrenergic receptor desensitization, downregulation of phospholamban and SERCA, and reduction in mitochondrial production of energy through downregulation of pyruvate dehydrogenase (PDH) activity [41]. As a results, it contributes to systolic and diastolic dysfunction and impaired contractile reserve [42]. Systemic inflammation in HFpEF induces oxidative and nitrosative stress that reduces NO bioavailability. As a result, the reduced activity of the PKG cardiovascular system induces myocardial hypertrophy and fibrosis, with increased cardiac and vascular stiffening [31,43]. A possible anti-inflammatory effect has been demonstrated for ACEI/ARBs and MRAs through a reduction in biomarkers (including CRP and cytokines like IL-6, TNF-alpha and INF-γ). In addition to that, SGLT2i also reduces markers of oxidative stress and ROS synthesis [44]. Although several systemic and target anti-inflammatory therapies have been tested in both HFrEF and HFpEF patients, the results are not encouraging. Colchicine demonstrated a reduction in inflammatory biomarkers without effects on exercise capacity, symptoms, hospitalizations, and mortality in HFrEF [45]; trials investigating its effects in HFpEF are ongoing [46]. TNF-alpha inhibitors, like etanercept or infliximab, failed to reduce mortality and hospitalization in HFrEF but they still have not been tested in HFpEF [47]. Finally, clinical trials investigating medications targeting IL-1 in HFpEF had discordant results. Anakinra (an IL-1 receptor antagonist) had positive results in terms of exercise tolerance improvement and reducing systemic inflammation in the DHART trial, but these results were not confirmed in the larger DHART2 trial [48]. Canakinumab (an anti IL-1β monoclonal antibody) was related to a dose-dependent reduction in heart failure hospitalizations and a composite of hospitalizations and HF-related mortality in a sub-analysis of the CANTOS trial [49] but there was no distinction between HFrEF and HFpEF in that study.

Pharmacological modulation of the NO pathway—through agents that restore NO bioavailability or stimulate downstream effectors like soluble guanylate cyclase (sGC)—represents a promising therapeutic avenue. However, it is crucial to recognize that NO bioavailability is highly susceptible to upstream perturbations. The substrate L-arginine is required for NO synthesis, while endogenous inhibitors of NOS, particularly asymmetric dimethylarginine (ADMA), can significantly reduce NO production. Additionally, oxidative stress and superoxide generation accelerate NO clearance, limiting its effectiveness and contributing to endothelial dysfunction [50,51].

High ADMA levels have been associated with worse clinical status, impaired renal function, and longer hospitalizations in patients admitted for acute decompensated HF [52]. Therefore, therapeutic strategies should not only target sGC activation but also aim to restore upstream NO production and reduce oxidative inactivation [53]. In HFrEF and HFpEF, impaired NOS activity due to substrate limitation, ADMA accumulation, and ROS-mediated NO degradation compounds the hemodynamic and structural consequences of HF. Approaches combining NO donors, antioxidants, or agents that reduce ADMA levels may enhance overall NO signaling and provide synergistic cardiovascular protection [54]. Furthermore, metabolic studies in advanced HF have shown that L-arginine and its derivatives correlate with exercise capacity, reinforcing the functional link between substrate availability, NO production, and clinical performance [55].

## 3. NO and Nitrate/Nitrite

Nitric oxide (NO) plays a pivotal role in cardiovascular homeostasis, regulating vascular tone, myocardial contractility, and organ perfusion. Endothelial dysfunction and reduced NO bioavailability are hallmarks of chronic heart failure (HF), contributing to elevated ventricular filling pressures and maladaptive remodeling [56].

Pharmacological NO donors, including organic nitrates, inorganic nitrites, and nitroprusside, have been used to restore NO signaling, reduce preload and afterload, and improve hemodynamics.

Organic nitrates (e.g., nitroglycerin, isosorbide dinitrate/mononitrate) should be considered the starting point of NO-targeted therapy in HF. They act as NO donors, undergoing enzymatic reduction to release NO, which activates soluble guanylate cyclase (sGC) to increase cGMP, leading to venodilation (reducing preload), modest arterial dilation (reducing afterload), improved cardiac output, and decreased mitral regurgitation and subendocardial ischemia [57,58]. However, chronic nitrate therapy is limited by tolerance, pseudo-tolerance (the rebound phenomenon), and resistance. Tolerance refers to the diminished hemodynamic response over time, while pseudo-tolerance or rebound phenomena involve neurohormonal counter-regulation, including increased sympathetic activity and renin–angiotensin system activation [59].

Resistance can extend to non-nitrate NO sources, reflecting broader impairments in endothelial NO signaling. Hydralazine is a direct arterial vasodilator that acts through mechanisms largely independent of the NO pathway. Its vasodilatory effect is thought to involve the opening of potassium channels in vascular smooth muscle cells and a reduction of intracellular calcium, leading to smooth muscle relaxation [33].

Clinically, the combination of ISDN with hydralazine, a direct arterial vasodilator acting largely independent of NO pathways, has demonstrated a significant reduction in long-term mortality in African American patients with HFrEF, whereas benefits in other racial groups remain less consistent [60]. It is crucial to distinguish the chemical forms: NO is rapidly oxidized to nitrite (NO_2_^−^), which can then be further oxidized to nitrate (NO_3_^−^), and these species are not interchangeable in terms of their pharmacodynamics [61].

Sodium nitroprusside (SNP) is an intravenous NO donor commonly used in acute decompensated HF for rapid preload and afterload reduction. SNP directly releases NO, activating sGC and increasing cGMP independently of enzymatic metabolism. It provides immediate hemodynamic improvement, especially in acute pulmonary edema or hypertensive crises and in acute decompensated heart failure [62].

In clinical practice, nitrates are primarily used in chronic HFrEF patients to alleviate symptoms related to congestion and ischemia, often in combination with hydralazine, especially in patients intolerant to or inadequately controlled by renin–angiotensin system inhibitors. The AHA/ACC guidelines recommend the combination of isosorbide dinitrate plus hydralazine as a Class I indication for African American patients with HFrEF (LVEF ≤40%) to improve survival and reduce hospitalizations [63]. The ESC guidelines position nitrates and hydralazine as alternative therapies for patients who cannot tolerate ACE inhibitors or ARNI but are less emphasized compared to newer agents [64]. For practical dosing, isosorbide dinitrate is typically started at 20 mg three times daily, titrated to 40 mg TID as tolerated, combined with hydralazine, starting at 25 mg three times daily and titrated to 50 mg TID [64]. Nitroglycerin is used acutely in intravenous form for rapid preload reduction but has limited chronic oral use due to tolerance.

In acute decompensated HF, intravenous nitrates remain a first-line vasodilator, providing rapid symptomatic relief by lowering left ventricular filling pressures and systemic vascular resistance, improving congestion without compromising perfusion when blood pressure permits. Inorganic nitrites (NO_2_^−^) and nitrates (NO_3_^−^) (e.g., dietary nitrate from beetroot or sodium nitrite) provide an alternative NO-generating pathway, bypassing dysfunctional endothelial NO synthase. Under conditions of hypoxia or acidosis—common in acute or exertional stress in heart failure—nitrite is reduced to NO, selectively improving perfusion in under-oxygenated tissues and augmenting cardiac output during exercise, particularly in HFpEF [65,66,67].

A recent systematic review and meta-analysis encompassing 14 trials (n ≈ 469) in HF and PH-LHD patients demonstrated that nitrate/nitrite therapy significantly improved resting hemodynamics (SBP, DBP, right atrial pressure) and modestly enhanced stroke volume and cardiac output during exercise, although improvements in peak oxygen consumption were less consistent [68]. These findings underscore the dual utility of nitrates/nitrites as chronic preload-reducing agents in stable HFrEF and as acute vasodilators in decompensated states, while highlighting the promise of nitric-oxide-releasing strategies that circumvent tolerance and endothelial dysfunction.

Pharmacological considerations are as follows:

- Organic nitrates primarily target venous capacitance vessels, reducing preload; arterial effects are modest. Chronic therapy requires nitrate-free intervals to mitigate tolerance.
- Nitrites offer selective vasodilation under hypoxic conditions and can act in both venous and arterial beds.
- SNP has a rapid onset and potent arterial and venous dilation but is limited to acute settings.
- Hydralazine provides arterial vasodilation independent of NO, complementing nitrate therapy.

Together, these NO donors set the pharmacological foundation for therapies that modulate the NO-sGC-cGMP pathway. While nitrates, nitrites, and SNP act upstream by delivering NO, sGC stimulators and activators (e.g., riociguat, vericiguat) directly increase cGMP independently of NO availability, providing an effective strategy in settings of severe endothelial dysfunction or oxidative stress.

## 4. Riociguat: A Role Beyond Pulmonary Hypertension?

Riociguat is an oral drug belonging to the soluble guanylate cyclase (sGC) stimulator class.

**Figure 2:**
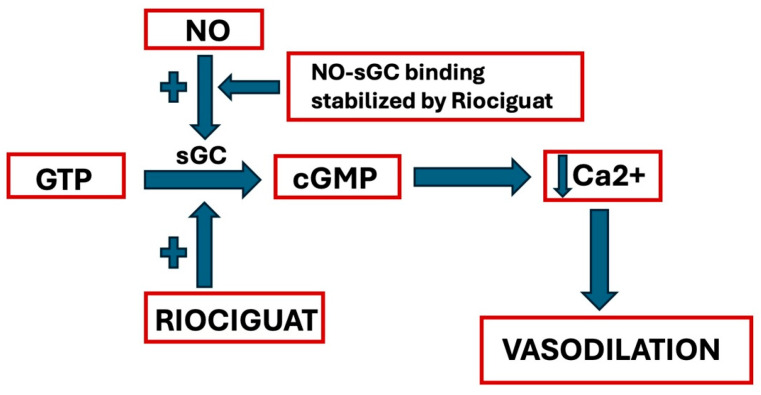
Riociguat’s mechanism of action (GTP = Guanosine Triphosphate; NO = Nitric Oxide; sGC = soluble Guanylyl Cyclase; cGMP = cyclic Guanosine Monophosphate). See text for explanation.

It has a double mechanism of action: it can directly stimulate sGC regardless of NO, but it can also increase sGC’s response to endogenous NO, stabilizing NO-sGC binding (Figure 2).

After administration, Riociguat is rapidly absorbed, reaching maximum blood concentration after 0.5–1.5 h. Its absorption is slightly influenced by food and it is metabolized by cytochromes P 450, mainly CYP1A1. Finally, it is eliminated by renal and fecal excretions. For this reason, Riociguat administration is not recommended in patients with severe hepatic impairment, with creatinine clearance lower than 15 mL/min, or on dialysis. Furthermore, to prevent hypotension, treatment initiation is not recommended if systolic blood pressure is lower than 95 mmHg, whereas if SBP is higher than 95 mmHg, during the treatment, it is recommended to uptitrate the dose till 2.5 mg three times daily [69].

Riociguat is one of the most important drugs in therapy of pulmonary arterial hypertension (PAH), a rare disease classified into five different categories and characterized by progressive pulmonary vascular remodeling. Indeed, sGC stimulators have demonstrated an important anti-inflammatory, antiproliferative, and antifibrotic effects in animal models.

Riociguat allows these patients to reach therapeutic objectives more frequently than other therapies. In PAH, endogenous NO synthesis may be reduced for coexistent endothelial dysfunction; therefore, phosphodiesterase-5 inhibition (PDE5i), mediated by PDE5 inhibitors, may be insufficient in cGMP signaling stimulation, which can be more effectively obtained with Riociguat, which can directly stimulate sGC [70].

One of the most important studies which demonstrate Riociguat’s efficacy in patients with PAH is the Pulmonary Arterial Hypertension Soluble Guanylate Cyclase–Stimulator Trial 1 (PATENT-1). After randomization to placebo or an orally adjusted dose, an increased six-minute walking distance was evident; it is an independent predictor of death and is considered the primary endpoint. There is also an improvement in time to clinical worsening, pulmonary vascular resistance (PVR), and right ventricular (RV) afterload with reduced NT-proBNP blood levels. Furthermore, these benefits were more evident in more advanced WHO functional classes (WHO-FC) [71].

Another important application is in chronic thromboembolic pulmonary hypertension (CTEPH), classified as type four of pulmonary hypertension, which is defined by chronic obstruction of pulmonary vessels mediated by organized thrombi. In this case, the gold-standard treatment is pulmonary endarterectomy, but not all patients are eligible and there are cases of persistent or recurrent pulmonary hypertension despite intervention.

Thereafter, the Chronic Thromboembolic Pulmonary Hypertension Soluble Guanylate Cyclase-Stimulator Trial 1 (CHEST-1), a randomized placebo-controlled study, evaluated Riociguat’s efficacy in patients with CTEPH ineligible for pulmonary endarterectomy or with persistent pulmonary hypertension after surgery. This trial demonstrated, in patients treated with Riociguat, a substantial improvement in six-minute walking distance (the primary endpoint of the study) and a reduction in PVR, which can also improve post-operative outcomes of patients suitable for surgery [72].

PAH is often associated with another widespread condition worsening its prognosis, namely HFpEF. For this reason, the haemoDYNAMIC trial (DYNAMIC), a randomized placebo-controlled study, evaluated the hemodynamic effects, safety, and tolerability of Riociguat in patients with PH-HFpEF. This trial demonstrated an improvement in cardiac output (CO, the primary outcome), considered a parameter representative of exercise capacity and of RV function. The CO increase may be favored by pulmonary vascular dilation, with an increased stroke volume mediated by Riociguat, and by positive remodeling effects on both ventricles.

A reduction in PVR, but without a significant improvement in NT-proBNP and quality of life, was also observed.

Finally, Riociguat was safe in most patients with PH-HFpEF, but without significant changes in clinical symptoms [73].

Riociguat has also been evaluated in patients with HFrEF, particularly in the context of combined post-capillary and pre-capillary pulmonary hypertension. The Left ventricular systolic dysfunction associated with pulmonary hypertension riociguat trial (LEPHT trial) was a multicenter, randomized, double-blind, placebo-controlled phase 2 study that investigated the hemodynamic and functional effects of riociguat in patients with symptomatic left ventricular systolic dysfunction (LVEF ≤ 40%) and mean pulmonary artery pressure ≥ 25 mmHg. Although the primary endpoint—change in mean pulmonary artery pressure (mPAP)—was not met, treatment with riociguat at the highest dose (2.0 mg TID) resulted in significant improvements in cardiac index, reductions in both pulmonary and systemic vascular resistance, and a trend toward improved six-minute walking distance and quality of life [74]. The duration and sample size may have been insufficient to detect clinically meaningful differences, and the short 16-week follow-up may not have allowed full hemodynamic effects to emerge. Dose escalation was also limited by systemic hypotension, restricting maximal pharmacologic impact [75].

Potential biases include regression to the mean in the placebo group—seen as unexpected mPAP reductions—and heterogeneity in patient background therapies and comorbidities across centers. In summary, riociguat’s failure in HFrEF likely stemmed from underpowered trial design, short follow-up, hypotension-limited dosing, and unexpected placebo responses, leaving its clinical utility in this population unproven. Table 1 provides a summary of the LEPHT trial.

Riociguat is recommended by the 2022 European Society of Cardiology/European Respiratory Society (ESC/ERS) Guidelines for pulmonary hypertension (PH) in patients with inoperable chronic thromboembolic pulmonary hypertension (CTEPH) or with persistent/recurrent PH after pulmonary endarterectomy (Class I, Level of Evidence B). It should not be combined with PDE5 inhibitors due to an increased risk of hypotension and lack of additional benefit [76]. The 2022 American College of Cardiology/American Heart Association/Heart Failure Society of America (AHA/ACC/HFSA) Guidelines for heart failure do not recommend Riociguat for routine use in HFrEF or HFpEF, given insufficient clinical evidence [77].

## 5. Vericiguat, a Non-Identical Twin and sGC Stimulator: Mission (Not) Impossible

Vericiguat is a novel sGC stimulator approved for the treatment of HFrEF.

In patients with heart failure and cardiovascular risk factors, reactive oxygen species produced by the endothelium reduce nitric oxide bioavailability. Nitric oxide binds to soluble guanylate cyclase (sGC), thereby stimulating the synthesis of intracellular cyclic guanosine monophosphate (cGMP), a key second messenger involved in the regulation of vascular tone, cardiac contractility, and remodeling [78].

Vericiguat directly stimulates sGC, activating the cGMP signaling pathway independently of nitric oxide (NO). In addition, it sensitizes sGC to NO by stabilizing NO binding at its receptor site. 

**Figure 3:**
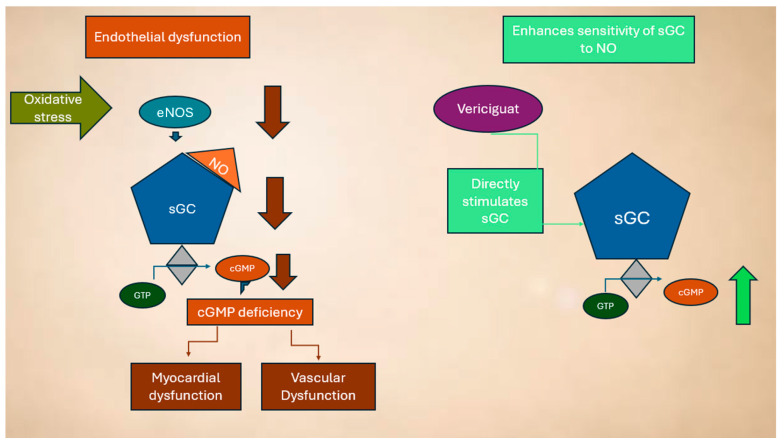
Dysregulation of the NO system in HF and mechanism of action of vericiguat (see text for explanation).

As a result, vericiguat restores the impaired NO-sGC-cGMP signaling pathway, even under conditions of low NO bioavailability and oxidative stress, ultimately leading to improvements in cardiovascular function [79], as shown in Figure 3.

The main clinical evidence on the use of vericiguat in HF is based on the Victoria trial. In this trial, the efficacy and safety of vericiguat were assessed in patients with a reduced ejection fraction and chronic HF with recent decompensation [80]. Patients were then randomly assigned, in a 1:1 ratio, to standard therapy + placebo and standard therapy + vericiguat at an increasing dose (2.5 mg > 5 mg > 10 mg). In this high-risk population, treatment with vericiguat on top of the standard of care resulted in a 10% relative reduction, compared to placebo, in the risk of the primary outcome of a composite of death from cardiovascular causes or first hospitalization for HF after a median treatment period of 10.8 months [79]. The main adverse events were symptomatic hypotension, syncope, and anemia [80]. No dosage adjustment of vericiguat is advised in patients with mild hepatic impairment (Child–Pugh A) or moderate hepatic impairment (Child–Pugh B). Moreover, no dosage adjustments are suggested in patients with eGFR ≥ 15 mL/min [78]. Therefore, the 2021 guidelines of the European Society of Cardiology recommend considering therapy with vericiguat when a patient with HFrEF is in New York Heart Association (NYHA) functional class II-IV and has developed an exacerbation of heart failure despite therapy with renin–angiotensin system antagonists, beta blockers, and antialdosterones (class IIb, level of evidence B). [64]. In this context, the VELOCITY study provided additional safety data on vericiguat initiation. It evaluated the tolerability of starting treatment directly at 5 mg daily, skipping the usual 2.5 mg lead-in dose. In this open-label trial, 106 patients with HFrEF were enrolled, and over 93% completed the two-week treatment period without moderate-to-severe symptomatic hypotension or therapy interruption. These results suggest that a simplified initiation strategy may be feasible in clinically stable patients already receiving optimal guideline-directed medical therapy. However, the study had some limitations, including a small sample size, short follow-up duration (14 days), and the exclusion of patients with low systolic blood pressure or significant comorbidities, which may limit generalizability to broader heart failure populations [81].

More recently, the VICTOR trial evaluated vericiguat in a population of clinically stable HFrEF patients (EF ≤ 40%) without recent HF worsening, on top of optimized guideline-directed medical therapy (GDMT). Contrary to VICTORIA, VICTOR did not meet its primary composite endpoint of cardiovascular death or first HF hospitalization (HR 0.93, *p* = 0.22) [82]. However, vericiguat was associated with a significant reduction in cardiovascular death (HR 0.83, 95% CI 0.71–0.97) and all-cause mortality (HR 0.84, 95% CI 0.74–0.97), whereas HF hospitalizations were not significantly reduced. These differences between VICTOR and VICTORIA can be explained by the baseline characteristics and risk profile of the enrolled populations. VICTOR included older, clinically stable patients with lower NT-proBNP levels and fewer NYHA class III–IV patients, resulting in a lower incidence of events and less room for improvement in hospitalizations. In contrast, VICTORIA enrolled patients with recent HF decompensation, higher NT-proBNP, and a more unstable clinical course, resulting in more events and a clearer signal in the primary composite endpoint. Physiologically, patients with recent decompensation exhibit a more severely impaired NO-sGC-cGMP pathway, providing greater “headroom” for vericiguat to exert beneficial effects, whereas in stable patients, the pathway is less compromised, so the main effect is observed on mortality rather than hospitalizations. A pooled analysis of VICTOR and VICTORIA data confirms that vericiguat reduces the composite endpoint, as well as cardiovascular and all-cause mortality, particularly in patients with NT-proBNP ≤ 6000 pg/mL, supporting the clinical relevance of patient selection and baseline risk [83].

Although both vericiguat and riociguat are sGC stimulators acting through the NO-sGC-cGMP pathway, their clinical development and target populations differ significantly, resulting in distinct therapeutic profiles. The VICTORIA trial evaluated vericiguat in patients with HFrEF who had experienced a recent episode of decompensation, such as hospitalization or intravenous diuretics. The trial included over 5000 patients and aimed to reduce hard clinical endpoints—namely, cardiovascular death or heart failure hospitalization. In contrast, the LEPHT trial assessed riociguat in a smaller cohort of 201 patients with HFrEF complicated by pulmonary hypertension (PH) due to left heart disease (Group 2 PH). The primary endpoint of LEPHT was hemodynamic, focusing on changes in mean pulmonary artery pressure (mPAP), with additional secondary endpoints including cardiac index, pulmonary and systemic vascular resistance, and quality of life. In Table 2, we provide a comparison of the main studies evaluating vericiguat and riociguat.

Vericiguat is recommended as an add-on therapy in patients with HFrEF who remain symptomatic after recent worsening of heart failure, despite guideline-directed medical therapy (GDMT). This includes patients already treated with a renin–angiotensin system inhibitor, a beta-blocker, and a mineralocorticoid receptor antagonist. The 2021 ESC Guidelines on heart failure recommend considering Vericiguat in NYHA class II–IV HFrEF patients with a recent worsening event, such as hospitalization or the need for IV diuretics (Class IIb, Level of Evidence B) [64]. The 2022 AHA/ACC/HFSA Guidelines also support its use in high-risk HFrEF patients with recent decompensation, defining it as reasonable to reduce cardiovascular death and HF hospitalization when added to foundational therapy (Class IIb recommendation) [77]. Vericiguat is generally well tolerated, though caution is advised in patients with baseline hypotension (systolic BP < 100 mmHg). In conclusion, recent data from Victor, combined with data from VICTORIA and LEPTH, tell us that the clinical efficacy of sGC stimulators depends strongly on patient characteristics and disease status, highlighting the need for careful selection of patients to maximize therapeutic benefit in HFrEF.

## 6. PDE5i from Pulmonary Arterial Hypertension to Heart Failure: A Leap into the Dark?

**Figure 4:**
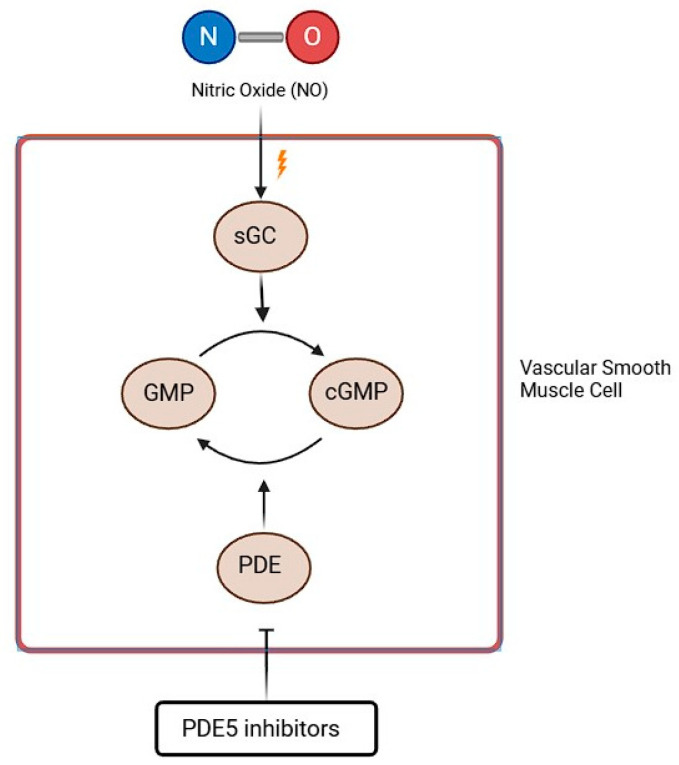
PDE5i mechanism of action (GTP = Guanosine Triphosphate; NO = Nitric Oxide; sGC = soluble Guanylyl Cyclase; cGMP = cyclic Guanosine Monophosphate). See text for explanation.

As reported above, NO is synthesized in endothelial cells by the enzyme NO synthase. It activates sGC, which leads to cGMP synthesis. cGMP’s effects include vasodilation, decrease in smooth muscle cell proliferation, and inhibition of platelet aggregation. These effects are mediated by cGMP kinase, which causes an upregulation of potassium (K^+^) channels and inhibition of calcium (Ca^++^) channels, thus resulting in a decreased intracellular Ca^++^ concentration. cGMP also activates myosin light chain phosphatase, which leads to a reduction in myosin phosphorylation and thus to pulmonary vasodilation [84]. Currently, 11 PDE family enzymes have been recognized, and each family differs in terms of substrate affinity, selectivity, or their regulatory mechanism. cGMP is selectively metabolized by PDE-5, PDE-6, and PDE-9 isoforms [85]. The mechanism of action of two classes of drugs—phosphodiesterase 5 inhibitors (PDE5i) and guanylate cyclase stimulators—is based on the pathway reported in Figure 4.

The main difference between the two agents is that PDE5i’s effects are dependent on NO synthesis, while guanylate cyclase stimulators are characterized by the possibility of directly stimulating sGC via a different binding site, independently of NO, whose bioavailability is reduced in PAH patients [86].

PDE5 inhibitors were introduced into the market as antianginal drugs and are now considered the first-line therapy of erectile dysfunction, which some studies have identified as an early sign of cardiovascular disease [87,88].

Furthermore, many preclinical and clinical trials have evaluated other possible uses of this class of drugs as regards their potential cardiovascular and anti-cancer benefits (including heart failure, myocardial ischemia/reperfusion injury, doxorubicin cardiotoxicity, ischemic and diabetic cardiomyopathy, cardiac hypertrophy, and Duchenne muscular dystrophy), with contrasting results [89]. PDE5i use in patients with heart failure with preserved ejection fraction, in the PhosphdiesteRasE-5 Inhibition to Improve CLinical Status and EXercise Capacity in Diastolic Heart Failure (RELAX) trial, showed no improvement in functional outcome and clinical status [66].

Type 5 is the predominant PDE isoform in the pulmonary vasculature. Conditions associated with PH are responsible for an upregulation of this enzyme [90]. It causes cGMP degradation, and thus PDE5i, contrasting with the action of PDE5, preventing or slowing cGMP metabolization and mediating the positive effects of endogenous NO. The PDE5i class includes three agents—sildenafil, tadalafil, and vardenafil—with only the first two approved for the treatment of pulmonary artery hypertension (PAH) [86].

Sildenafil is used at a dose of 20 mg three times a day and is also available in 10 mg/mL as an oral suspension. The use of Sildenafil in PAH was first evaluated by the Sildenafil Use in Pulmonary arterial hypertension (SUPER) trials. SUPER-1 was a double-blind, placebo-controlled study that enrolled 278 patients with treatment-naïve symptomatic PAH. These patients were divided into two groups: one group was assigned to sildenafil (20, 40, or 80 mg) orally three times daily and the other one was assigned to placebo. After 12 weeks, the authors found out that the distance walked in the six-minute walking test (6MWT) increased from the baseline regardless of sildenafil dose. Furthermore, all sildenafil doses caused a reduction in mean pulmonary artery pressure and improved the WHO functional class. Side effects were flushing, dyspepsia, and diarrhea. No difference in the incidence of clinical worsening was found between the two groups [90]

Tadalafil is another medication belonging to the PDE5i class. It is used at a dose of 20 mg once a day. Tadalafil use was evaluated in the Pulmonary arterial HypertensIon and ResponSe to Tadalafil (PHIRST) trials. PHIRST-1 was a 16-week, double-blind, placebo-controlled clinical study that enrolled 405 patients with symptomatic PAH. These patients were randomized into two groups: placebo or four doses of tadalafil—2.5, 10, 20, or 40 mg—orally once daily. According to the results of the study, Tadalafil managed to improve the distance in the 6MWT in a dose-dependent manner, but only the 40 mg dose reached the prespecified level of statistical significance (*p* < 0.01). Better results were achieved in patients that were not already treated with Bosentan, an endothelin receptor antagonist. Furthermore, tadalafil 40 mg delayed clinical worsening and improved health-related quality of life. However, changes in WHO functional class were not statistically significant. The most frequent adverse effects due to tadalafil use were headache, myalgia, and flushing [91].

According to a more recent study, PDE5i might also improve survival in responder patients with interstitial lung disease (ILD) and severe PH, with the smaller survival advantage in patients with right ventricular dysfunction at presentation. Based on these results, the 2022 ESC/ERS guidelines made a conditional recommendation, based on very low-quality evidence, that PDE5i could be considered in ILD patients with severe PH, specifically in the context of individualized decision-making and in experienced expert centers [92].

While PDE5i showed no advantageous effects in patients affected by pulmonary hypertension secondary to lung disease or chronic thromboembolic disease, some benefits were found for patients with pulmonary hypertension secondary to left heart disease, although it is unclear in which type of left heart disease [84].

In heart failure, especially in the presence of endothelial dysfunction and increased oxidative stress, endogenous NO availability is reduced, leading to impaired vasodilation, increased vascular resistance, and maladaptive remodeling. By preserving intracellular cGMP levels, PDE5i promotes vasorelaxation, reduces right and left ventricular afterload, and exerts anti-hypertrophic and anti-fibrotic effects at the myocardial level [17,93,94]. In HFrEF, particularly in patients with concomitant PH, multiple studies have demonstrated favorable hemodynamic and functional effects of PDE5 inhibition. These include reductions in mean pulmonary artery pressure (mPAP), pulmonary vascular resistance (PVR), and right atrial pressure, along with improvements in cardiac output, right ventricular ejection fraction (RVEF), and exercise tolerance [95,96].

Notably, randomized controlled trials such as those by Guazzi et al. demonstrated improvements in left ventricular diastolic function, reduced left ventricular mass index, and enhanced exercise capacity after chronic sildenafil administration in HFrEF patients [97]. A meta-analysis involving over 900 heart failure patients confirmed consistent benefits in peak VO_2_, 6 min walk distance (6MWD), and left ventricular ejection fraction (LVEF), along with a significant reduction in the composite endpoint of death or heart failure hospitalization (OR 0.28, *p* = 0.03) [98].

However, in HFpEF, evidence remains inconsistent; the RELAX trial showed no significant benefit of sildenafil on exercise capacity or clinical outcomes, possibly due to an unfavorable impact on ventricular contractility offsetting vascular effect [99,100]. The Sildenafil in Heart Failure (SilHF trial) in HFrEF patients with PH did not confirm clinical benefits, emphasizing the need for refined patient selection and phenotyping [101].

In advanced heart failure, a retrospective case–control study examined the effects of long-term sildenafil therapy in patients with advanced systolic heart failure and severe pre-capillary pulmonary hypertension. The results indicated that sildenafil significantly reduced pulmonary vascular resistance and transpulmonary gradient, increased cardiac output, and improved New York Heart Association (NYHA) functional class [102]. However, its use in patients with advanced or decompensated heart failure requires caution. Concerns include the risk of hypotension, especially when combined with other vasodilators. Additionally, the safety and efficacy of PDE5i in acute cardiac dysfunction or advanced decompensated heart failure have not been well established [103].

Overall, while PDE5i displays a promising mechanistic rationale and beneficial effects in selected HFrEF populations, further large-scale, phenotype-specific trials are required to establish its role in HF management. Despite the promising pathophysiological rationale and favorable hemodynamic effects observed with PDE5i agents in selected HF populations, these agents have not been widely adopted in routine clinical practice nor incorporated into major HF management guidelines. Several factors contribute to this cautious stance. Large-scale, adequately powered randomized controlled trials demonstrating definitive improvements in hard clinical endpoints such as mortality and heart failure hospitalizations are lacking. Most existing studies have relatively small sample sizes, short follow-up durations, and heterogeneous patient populations, limiting generalizability and statistical power. Major guidelines such as the 2022 ESC Heart Failure Guidelines and the 2023 ACC/AHA/HFSA Focused Update do not currently recommend PDE5i for HF treatment outside of pulmonary hypertension indications, reflecting insufficient evidence to support broad clinical application.

Table 3 reports the main studies evaluating the use of PDE5i in HF patients.

In pulmonary arterial hypertension (PAH), PDE5 inhibitors (sildenafil, tadalafil) are recommended as first-line therapy by both ESC/ERS and AHA/ACC guidelines (Class I, Level A), due to their efficacy in improving exercise capacity, symptoms, and hemodynamics [76,77]. In heart failure (HFrEF or HFpEF), PDE5i agents are not recommended for routine use outside of PAH. The 2022 ESC Heart Failure Guidelines and the 2022 AHA/ACC/HFSA Update do not support PDE5i for primary HF treatment due to inconsistent evidence and lack of outcome benefit in large trials like Phosphodiesterase-5 Inhibition to Improve Clinical Status and Exercise Capacity in Heart Failure with Preserved Ejection Fraction (the RELAX trial) and SilHF [64,101].

In selected patients with HF and PAH, particularly those with preserved ejection fraction or advanced disease, PDE5i agents may offer symptomatic and hemodynamic benefits. However, evidence is mainly from case reports and small observational studies, and their use should be individualized and carefully monitored.

## 7. Nebivolol and NO

According to 2021 ESC guidelines for the diagnosis and treatment of HF, β-blockers are disease-modifying drugs that have a significant impact on the long-term prognosis in patients with left ventricular dysfunction [64]. The protection of the myocardium against the toxic and proarrhythmic effect of an excess of catecholamines, the reduced myocardial oxygen consumption, and the reduction in heart rate justify the use of β-blockers in the treatment of patients with heart failure. Bisoprolol, carvedilol, metoprolol, and nebivolol are the four β-blockers indicated in this setting.

Nebivolol is a third-generation β1-receptor blocker drug that is used in the treatment of systemic arterial hypertension and heart failure. In addition to a high cardioselectivity through the antagonistic action on the β1-receptor, thus determining a negative chronotropic effect, nebivolol exerts a vasodilatory effect mediated by β3-receptor stimulation, which induces endothelial nitric oxide (NO) synthesis [105]. This β3-mediated vasodilatory effect of nebivolol has also stimulated interest in developing selective β3-agonists as potential therapies for HF. β3-agonists such as mirabegron represent a novel therapeutic avenue distinct from traditional β1- and β2-targeting agents. They reduce oxidative stress and sodium overload in cardiomyocytes, improving left ventricular function. A 2016 randomized invasive clinical trial evaluated the hemodynamic effects of mirabegron in 22 patients with advanced HF (HFrEF, EF < 35%, NYHA class III–IV). Patients received mirabegron for one week in addition to standard therapy. Treatment significantly increased cardiac index and reduced pulmonary vascular resistance compared to placebo, suggesting improved cardiac function [106]. More recently, the Beta3-LVH trial investigated mirabegron in patients with mild HF (NYHA class I–II) over 12 months. While no significant differences were observed in left ventricular mass index (LVMI) or diastolic function between mirabegron and placebo, exploratory analyses suggest potential benefits in advanced or end-stage HF [107]. Importantly, nebivolol has been shown to induce NO production within the heart, contributing to its distinctive hemodynamic profile compared to other β-blockers. Its use in patients with systemic arterial hypertension is associated with preservation of cardiac output and reduced peripheral resistance, which may translate into lower mortality and hospitalization rates in HF patients. Nebivolol also stimulates inducible nitric oxide synthase (iNOS), which is upregulated in HF and may play a protective role. The use of nebivolol in the treatment of patients with systemic arterial hypertension is associated with the preservation of cardiac output and the reduction in peripheral resistance, contributing to reducing mortality and the rate of hospitalization in patients with heart failure. The induction of NO synthesis could explain the better hemodynamic profile of nebivolol compared to other β-blockers, since NO improves coronary blood flow and reduces cardiac hypertrophy. Additionally, nebivolol stimulates the activity of iNOS, which is increased in heart failure and may have a protective role [108]. Over the years, several studies have evaluated the effectiveness of nebivolol and β-blockers in general in the treatment of heart failure.

The SENIORS (Study of effects of nebivolol intervention on outcomes and rehospitalisation in seniors with heart failure) evaluated the effect of nebivolol compared with placebo on morbidity and mortality in elderly patients with heart failure. It enrolled 2128 patients, with a follow-up period of up to 40 months. The primary endpoints were represented by all-cause mortality or cardiovascular hospital admission. Patients were randomized to the β-blocker nebivolol (uptitrated to 10 mg) or placebo. The difference between SENIORS and earlier β-blocker trials was represented by the average age (76 years vs. 61 years). Among elderly patients with HF regardless of ejection fraction, treatment with the β-blocker nebivolol was associated with a reduction in the primary endpoint of all-cause mortality and admission for cardiovascular events compared with placebo [109]. In this regard, the SENIORS trial’s post hoc analyses revealed that nebivolol may reduce hospitalizations and improve symptoms in elderly heart failure patients, including those with preserved ejection fraction [110].

A recent review highlighted that β-blockers are often not prescribed or underdosed for fear of side effects and comorbidities. A possible impairment in pulmonary function, hypoglycemia, and erectile dysfunction should not limit the use of β-blockers but only require closer dose monitoring. Knowledge of drug–disease interactions is important to guide prescription and direct the choice of a specific agent in individual patients. Nebivolol is indicated as a second choice, after bisoprolol, in patients with chronic obstructive pulmonary disease (COPD) and erectile dysfunction and after carvedilol in patients with diabetes mellitus [111]. Moreover, compared to other β-blockers, nebivolol has demonstrated superiority in patients with peripheral arterial disease (PAD). In addition to its coronary vasodilating activity through the activation of the NO pathway mediated by the stimulation of inositol metabolism, which manifests itself with a dose-dependent effect and is related to the particular lipophilicity of the molecule, which would promote its accumulation in the endothelium, nebivolol also has an antithrombotic effect, modulating the activity of the endothelium through the prostaglandin PGI2. Through an experimental model that involved the formation of thrombi in conditions of extracorporeal circulation, the antithrombotic effect of nebivolol was demonstrated, mediated by the activation of β2-receptors and inhibited by COX-2 inhibitor drugs such as indomethacin [112].

A recent study investigated the role of nebivolol in cardiorenal syndrome. Through the myocardial infarction model developed, it demonstrated nebivolol’s enhancement of NO-mediated effects such as the prevention of nitrosative damages through the reduction in the activity of iNOS, preservation of the glomerular filtration rate through neuronal NOS (nNOS), and restoration of eNOS in the late period of MI. Therefore, nebivolol has real efficacy in preventing both subclinical and clinical nephropathy [113]. In patients with heart failure and preserved ejection fraction, iodine-123 metaiodobenzylguanidine (123I-MIBG) scintigraphic parameters and peak VO2 in the cardiopulmonary exercise test (CPET) represent important prognostic markers. Based on its vasodilating properties, the use of nebivolol in improving these parameters was evaluated. Despite better control in systolic blood pressure and heart rate during rest and exercise, nebivolol was not effective in improving peak VO2 and 123I-MIBG scintigraphic parameters. Moreover, targeted studies demonstrate nebivolol’s capacity to improve endothelial function and reduce oxidative stress in HFpEF populations, suggesting a potential reversal of key pathological processes [114]. The hypothesis of its ineffectiveness on functional capacity could be linked to the lack of an effect on adrenergic activity [115].

Nebivolol, a β1-selective blocker with nitric oxide-mediated vasodilatory effects, is recommended in heart failure with HFrEF based on the ESC 2021 guidelines due to its proven reduction in mortality and cardiovascular hospitalizations, particularly in elderly patients [109]. It is favored in patients with comorbidities such as chronic obstructive pulmonary disease (COPD) and erectile dysfunction due to its better safety and tolerability profile compared to other β-blockers [116]. Evidence in HFpEF remains limited and inconsistent; however, smaller clinical studies and case reports suggest nebivolol may improve endothelial function and reduce oxidative stress, potentially benefiting select HFpEF patients. These effects are thought to be mediated by increased NO bioavailability through eNOS activation and antioxidant properties demonstrated in preclinical models.

## 8. Conclusions

Nitric oxide plays a pivotal role in vascular tone regulation, organ perfusion, and endothelial function. Endothelial dysfunction and reduced NO bioavailability contribute significantly to disease progression and poor prognosis in patients with pulmonary hypertension, heart failure, and chronic kidney disease. Pharmacological agents targeting the NO-cGMP pathway—including the “non-identical twin” sGC stimulators (riociguat, vericiguat), phosphodiesterase-5 inhibitors (sildenafil, tadalafil), and the β-blocker nebivolol—offer targeted strategies to restore NO signaling and improve clinical outcomes. Riociguat and vericiguat directly enhance cGMP levels, bypassing NO deficiency, whereas PDE5 inhibitors prolong cGMP action, and nebivolol stimulates endogenous NO release, which is particularly advantageous in patients with elevated oxidative stress and arterial stiffness. Alternative NO sources, including inorganic nitrites (NO_2_^−^) and nitrates (NO_3_^−^), act via enzymatic and non-enzymatic reduction pathways, bypassing endothelial NO synthase, and provide additional modulation of the NO system. These agents can improve perfusion under hypoxic or acidic conditions, augment cardiac output during exercise, and complement chronic NO-targeted therapies. Sodium nitroprusside, a direct NO donor, offers rapid vasodilation in acute settings such as pulmonary edema or hypertensive crises, though its chronic use is limited by cyanide toxicity. Integrating these alternative NO sources into HF management highlights the potential for layered, flexible therapeutic strategies.

In complex HF phenotypes—such as HFrEF with CKD or pulmonary hypertension—NO-sGC-cGMP modulators can serve as early adjuncts or even initial therapeutic steps, especially when GDMT is limited by hypotension or renal impairment. By restoring protective intracellular signaling, these agents facilitate diuresis, relieve congestion, and improve outcomes in challenging populations. Recent trial data refine our understanding of vericiguat’s clinical profile. In VICTORIA, high-risk patients with recent HF worsening experienced a 10% relative reduction in the composite endpoint of cardiovascular death or HF hospitalization. In contrast, VICTOR enrolled more stable, ambulatory patients with lower NT-proBNP and predominantly NYHA II status. The primary composite endpoint was not significantly reduced (HR 0.93; *p* = 0.22), although nominal reductions in cardiovascular and all-cause mortality were observed. This divergence reflects differences in patient risk, disease activity, and baseline therapy: VICTORIA patients had higher event rates and recent decompensation, providing more “headroom” to detect benefit, whereas VICTOR patients had optimized GDMT and fewer events, limiting detectable impact on the composite. Pooled analyses of both trials confirm that vericiguat reduces cardiovascular events and HF hospitalizations, particularly in patients with NT-proBNP ≤ 6000 pg/mL, demonstrating that patient selection is key to maximizing benefit.

The lessons gleaned from the studies on Vericiguat and Riociguat extend beyond the individual drug profiles. They underscore the critical importance of a thorough understanding of the intricate NO pathway and the judicious selection of patient populations most likely to derive benefit from targeted therapies.

Future research should focus on optimizing NO–cGMP modulation in advanced CKD and HF patients, exploring novel sGC stimulators with improved safety profiles, combinatory regimens (e.g., with SGLT2 inhibitors) for synergistic cardiorenal protection, and identifying patient subgroups most likely to benefit without undue hypotensive risk.

In conclusion, pharmacological modulators of the NO pathway—including sGC stimulators/activators, PDE5 inhibitors, nebivolol, and alternative NO sources such as nitrites, nitrates, and acute donors like nitroprusside—represent a cohesive framework to translate molecular understanding into tangible clinical benefits. Moreover, the discussion of renal implications, tolerance limitations, and complementary NO sources emphasizes the relevance of personalized, cardiorenal-focused strategies for HF management.

## Figures and Tables

**Table 1:** LEPHT trial.

Inclusion Criteria	Exclusion Criteria
Age between 18 and 80 years	LVEF < 50%
Confirmed PH-HFpEF diagnosis (Dana Point Group 2.2)	Significant valvular heart disease
WHO functional class II–IV	Severe obstructive or restrictive lung disease
LVEF ≥ 50% (preserved systolic function)	Severe hepatic or renal dysfunction
mPAP ≥ 25 mmHg at rest (right heart catheterization)	Recent acute coronary syndrome or stroke (<3 months)
PAWP > 15 mmHg at rest	Use of pulmonary vasodilators within 30 days
Stable heart failure and antihypertensive therapy ≥ 30 d	Pregnancy or breastfeeding
Stable diuretic therapy ≥ 7 days	Inability to comply with study procedures
Right heart catheterization within prior 12 weeks	History of pulmonary embolism or chronic thromboembolic PH (CTEPH)
(Optional) cardiac MRI within 12 weeks	Known hypersensitivity to Riociguat or study drug components
Signed informed consent	

**Table 2:** Comparison of studies on vericiguat and riociguat.

Characteristic	VICTORIA	LEPTH
Study Phase	Phase III, randomized, double-blind, placebo-controlled	Phase IIb, randomized, double-blind, placebo-controlled
Sample Size	5050 patients	201 patients
Population	HFrEF (EF ≤ 45%), recent worsening (hospitalization/IV diuretics)	HFrEF (EF ≤ 40%) + post-capillary PH (mPAP ≥ 25 mmHg)
Follow-up Duration	Median 10.8 months	16 weeks
Primary Endpoint	CV death or HF hospitalization	Change in mPAP from baseline
Primary Endpoint Result	Significant risk reduction (HR 0.90, *p* = 0.02)	Non-significant change in mPAP (*p* = 0.10)
Cardiac Index	Not a primary focus; unchanged	Significant increase (+0.4 L/min/m^2^, *p* = 0.0001)
Pulmonary/Systemic Vascular Resistance	Not assessed	Significant reductions (PVR *p* = 0.033; SVR *p* = 0.0002)
NT-proBNP	Median ~2800 pg/mL	Median ~1300 pg/mL (estimated from published data)
Background HF Therapy	Optimized GDMT (β-blockers, ACEi/ARB/ARNi, MRA, CRT/ICD)	Not standardized or detailed

**Table 3:** List of available studies on PDE5i use in HF.

Study	Population	Sample Size	Mean Age (years)	Sex (% Male)	LVEF (%)	NYHA Class	PH	Key Inclusion Criteria	Treatment Duration	Primary Endpoint
Guazzi et al. [97]	HFrEF with PH	45	61 ± 10	84%	32 ± 5	II–III	Yes	Stable HFrEF, PASP > 40 mmHg	12 months	LV diastolic function, peak VO_2_
Lewis et al. [95]	HFrEF with secondary PH	34	59 ± 11	88%	29 ± 6	II–IV	Yes	HFrEF with PASP ≥ 40 mmHg	12 weeks	Pulmonary hemodynamics, exercise capacity
SilHF trial [101]	HFrEF with PH	69	63 ± 9	79%	30 ± 6	II–III	Yes	HFrEF with PASP ≥ 40 mmHg	24 weeks	Symptoms, 6MWT, PASP
RELAX trial [99]	HFpEF	216	69 ± 10	48%	60 ± 7	II–III	Mixed	HFpEF diagnosis (EF ≥ 50%), elevated NT-proBNP	24 weeks	Exercise capacity, clinical status
Belyavskiy et al., 2020 [104]	HFpEF with combined PH	50	65 ± 12	52%	56 ± 6	II–III	Yes	HFpEF with combined pre/post-capillary PH	6 months	6MWT, echo, hemodynamics

## Data Availability

The data from this manuscript are derived from publicly available published clinical trial and study results.
